# A Model-Based Joint Feature Fusion framework for limited-view photoacoustic tomography reconstruction

**DOI:** 10.1016/j.pacs.2026.100886

**Published:** 2026-09-22

**Authors:** Biru Zhang, Jiankun Wang, Max Q.-H. Meng

**Affiliations:** a Shenzhen Key Laboratory of Robotics Perception and Intelligence, Department of Electronic and Electrical Engineering, Southern University of Science and Technology, Shenzhen, China; b Jiaxing Research Institute, Southern University of Science and Technology, Jiaxing, Zhejiang, China

**Keywords:** Photoacoustic tomography, Reconstruction, Limited-view, Model-based learning

## Abstract

In photoacoustic tomography (PAT) reconstruction, imaging systems with limited-view detection geometries capture acoustic signals over restricted angular ranges, which results in reconstruction artifacts and degraded image quality. Model-based iterative methods can effectively compensate for limited-view artifacts, but their iterative optimization procedures remain computationally demanding. While model-based learning methods leverage neural networks to substantially improve reconstruction quality and accelerate convergence, they remain computationally expensive due to repeated evaluations of the physical forward model. Consequently, their applicability in real-time or near-real-time scenarios is limited. To overcome this constraint, this study proposes a novel overall framework: the Model-Based Joint Feature Fusion (MB-JFF) framework, which is developed under the assumption of a homogeneous and lossless acoustic medium. This framework combines the powerful feature extraction capabilities of a joint feature fusion module with the priors embedded in a model-based module. The joint feature fusion module effectively fuses information from both the original sensor data and the model-driven residual sensor data. By integrating these two modules, the proposed method effectively combines the strengths of deep learning with guidance from the forward model, achieving high-quality reconstruction with only a single iteration. Furthermore, we introduce a learned hybrid forward operator that serves as a geometry-informed surrogate forward model within the MB-JFF framework. Through leveraging geometric mapping and a learned wave propagation refiner, this operator rapidly predicts sensor data that closely approximate those obtained from full-wave numerical solvers, while substantially reducing the associated computational cost. Comparative experimental results validate the exceptional performance of the MB-JFF framework. Our proposed approach demonstrates superior reconstruction quality while achieving a notable reduction in computational time compared with other model-based reconstruction methods.

## 1 Introduction

Photoacoustic tomography (PAT) represents an innovative biomedical imaging modality that synergistically integrates optical excitation with acoustic detection [1]. This technique leverages the photoacoustic effect, which is triggered by pulsed laser irradiation. The laser irradiation induces optical absorption in endogenous chromophores (such as hemoglobin and melanin) or exogenous contrast agents [2], [3]. Then, the absorbed optical energy is rapidly converted into heat, leading to a transient temperature rise. This localized heating induces thermoelastic expansion of the tissue, which in turn generates ultrasonic waves (photoacoustic signals) that encode both structural and functional information about the tissue [4], [5]. These signals are then detected by the transducer and subsequently reconstructed into a diagnostic image. A significant advantage of this approach stems from the fact that ultrasonic scattering in tissue is approximately three orders of magnitude weaker than optical scattering [1]. This enables PAT to achieve greater imaging depth than traditional optical imaging, while maintaining high optical absorption contrast through optical excitation. Furthermore, since the amplitude of the photoacoustic signal correlates directly with the optical absorption coefficient, PAT is particularly well-suited for visualizing optically absorbing biological structures, making it a promising non-invasive, non-ionizing approach for cancer diagnosis and monitoring [6], [7], [8].

The process of converting photoacoustic (PA) sensor data into images is referred to as PAT reconstruction. In PAT reconstruction, acoustic waves generated within the tissue propagate omnidirectionally. As illustrated in Fig. 1, a circular transducer array can provide full-view acquisition of the acoustic signals. In contrast, linear and curved transducer arrays do not achieve full 360° angular coverage and can only receive signals from a limited range of directions, resulting in missing information from unobserved views [9], [10]. This incomplete angular sampling, known as the limited-view problem, leads to reconstruction artifacts and loss of structural detail in the reconstructed images [11], [12], [13].

**Fig. 1:**
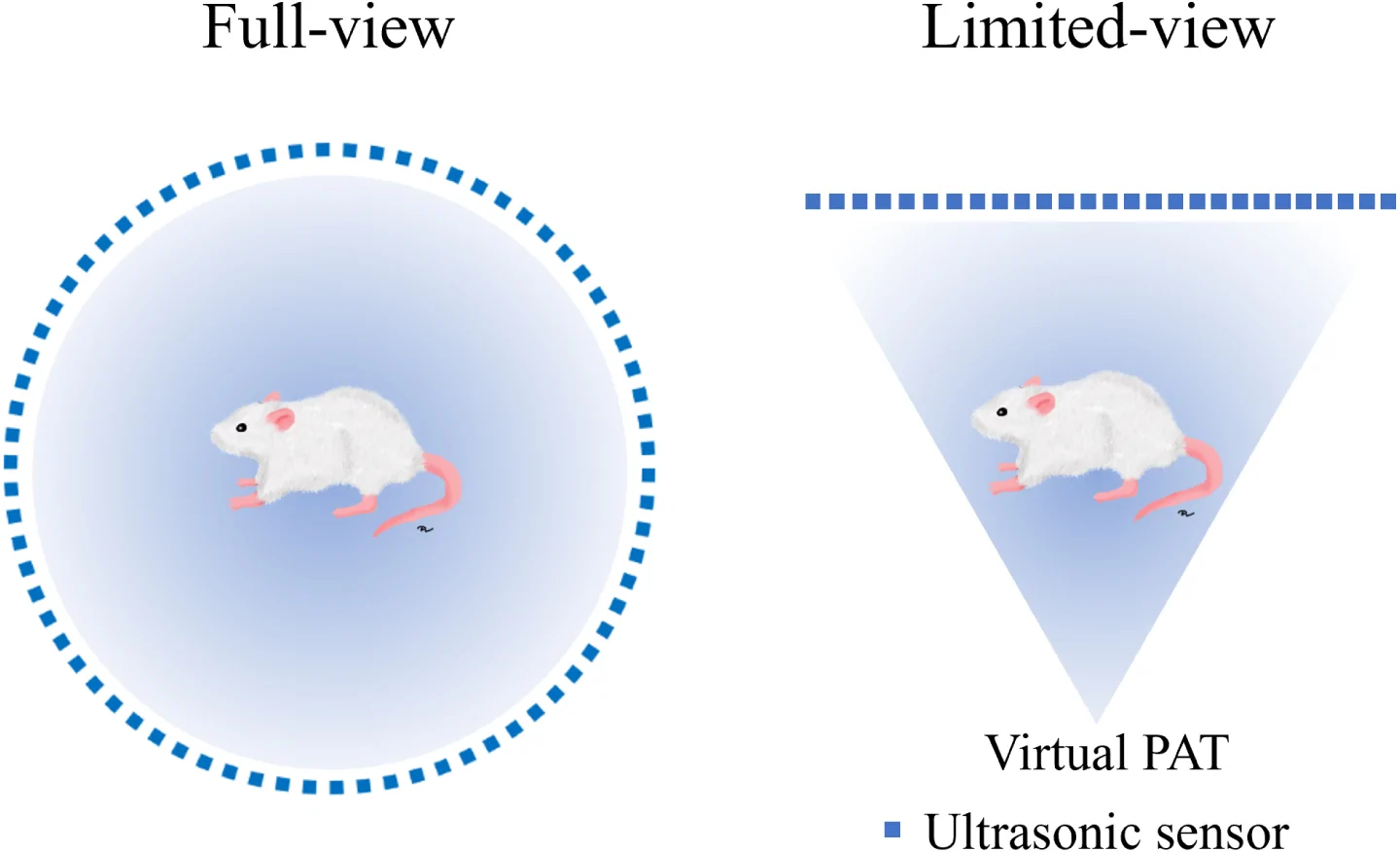
Illustration of the limited-view problem in PAT.

To address the limited-view problem in PAT reconstruction, this paper makes the following key contributions:

1. This study proposes a novel overall framework called the model-based joint feature fusion framework, which leverages feature fusion and a joint network to better exploit the information from both the original sinogram and the model-driven residual sinogram. This framework is capable of improving reconstruction quality, enabling a high-quality image to be produced with a single iteration.
2. We design a new learned hybrid forward operator as a geometry-informed surrogate forward model within the model-based joint feature fusion (MB-JFF) framework. This operator combines a geometric mapping with a learned wave propagation refiner to rapidly generate high-fidelity sinograms for the subsequent generation of model-driven residual information.
3. Through the combination of a novel framework and a new learned hybrid forward operator strategy, we are able to substantially reduce computational time while still achieving high-quality reconstructed images. The effectiveness of the proposed method is demonstrated in the comparative experiments.

## 2 Related work

Deterministic methods for reconstructing PAT images can generally be divided into three main categories: traditional algorithms, deep learning-based methods, and model-based learning methods. Among traditional algorithms, back-projection (BP), time-reversal (TR), and model-based iterative (MBI) methods are commonly employed. BP reconstructs images by back-projecting the detected photoacoustic signals along the assumed spherical wavefront [14], [15], whereas TR numerically simulates the time-reversed propagation of acoustic waves in the medium [16], [17]. Moreover, MBI methods formulate image reconstruction as a least-squares optimization problem constrained by the forward propagation of the PA wave [18], [19]. While MBI methods can partially compensate for limited-view artifacts through regularization, their performance depends on the choice of regularizer and the tuning of its associated parameters. Overall, recovering missing information under severely restricted angular sampling conditions remains challenging for these traditional approaches. Their reconstruction quality is still constrained by the inherent data deficiency associated with limited-view PA signal acquisition.

Deep learning-based methods can learn complex mappings from incomplete data. By leveraging statistical priors learned from training data, these approaches can implicitly infer missing information, enabling improved image quality under limited-view acquisition. Existing deep learning approaches for PAT reconstruction are broadly grouped into four categories: (1) end-to-end networks that directly map PA sinograms to images [20], [21], [22], [23]; (2) preprocessing networks that only refine sensor data [11], [24]; (3) post-processing networks that enhance initially reconstructed images [25], [26], [27], [28]; and (4) hybrid-input networks that jointly utilize both raw sensor data and preliminary reconstructions as inputs [13], [29], [30]. These learning-based approaches remain purely data-driven, and their reconstruction quality ultimately depends on the information from the training dataset.

As a hybrid paradigm, model-based learning (MBL) integrates MBI optimization with deep learning, leveraging forward model guidance and learned implicit priors to improve image quality. In MBL, neural networks implicitly learn parameters that are traditionally handcrafted in MBI approaches [31], [32]. Beyond parameter learning, some networks can also generate enhanced intermediate solutions as inputs for the gradient descent step [33], [34], [35]. Although high-quality images can be obtained, MBL faces computational challenges: the forward operator requires tedious physical simulation, and the iterative reconstruction procedure further increases computational cost.

Among existing forward operator solvers, high-fidelity full-wave methods [36], such as the k-space pseudospectral time-domain (k-space PSTD) method [37], [38], are widely adopted due to their high numerical accuracy and physical fidelity. However, these methods typically require computationally expensive stepwise wave propagation simulations. Alternatively, matrix-based approaches approximate acoustic propagation as a linear system and compute the sinogram via matrix–vector multiplication [39]. To improve computational efficiency, such methods often employ simplified physical models, which reduce modeling fidelity compared with full-wave simulations. Recently, learned surrogate forward models have emerged as a promising alternative to conventional numerical forward solvers for accelerating MBL pipelines. Notably, a recent study replaced the traditional forward operator with a Fourier Neural Operator (FNO) [40] to reduce computational time. However, FNO learns a globally coupled spatiotemporal operator and predicts the complete acoustic wave field across the entire spatial domain and all time steps simultaneously. As a result, its computational and memory complexity scale rapidly with the temporal dimension, limiting its applicability to scenarios with a small total number of time steps.

It should also be noted that the data deficiency makes limited-view PAT reconstruction an ill-posed inverse problem, because the missing angular information cannot, in general, be uniquely recovered from the measured data alone. Therefore, beyond deterministic methods that aim to obtain a single high-quality image estimate, probabilistic methods provide an important complementary direction by estimating uncertainty or posterior distributions of images consistent with the measured data [41], [42].

To achieve fast and high-quality limited-view PAT reconstruction, this study proposes a novel deterministic framework, called MB-JFF, and a new learned hybrid forward operator strategy. The proposed method integrates forward-model guidance with deep-learning priors via a joint network architecture, as detailed in the following section.

## 3 Preliminaries

### 3.1 Model-based iterative reconstruction

The MBI method demonstrates superior performance in limited-view PAT reconstruction by solving an optimization problem to approximate the real solution. It can be mathematically formulated as:

$$\overset{ˆ}{x}=\arg\underset{x}{\min}\left\{\frac{1}{2}{\left\|A\left(x\right)-y\right\|}_{2}^{2}+\lambda R\left(x\right)\right\},$$

where x denotes the reconstructed initial pressure distribution, $\overset{ˆ}{x}$ represents the optimized solution, and y denotes the measured sinogram. A(⋅) denotes the forward operator that models the photoacoustic wave propagation from the initial pressure distribution to the detected acoustic signals. R(⋅) is the regularization term designed to suppress noise and artifacts, and λ is the regularization parameter that balances the data fidelity term and the regularization term. The term ${\left\|A\left(x\right)-y\right\|}_{2}^{2}$ corresponds to the data fidelity term, which measures the discrepancy between the measured sinogram and the predicted measurements generated by the forward operator.

This optimization problem is typically solved iteratively using a gradient descent procedure. One key limitation of MBI methods is the high computational cost associated with the iterative optimization process. Another major limitation is that both the regularization term and its corresponding weighting parameter require manual design and tuning, which directly impacts the quality of the reconstructed images. To improve reconstruction performance, several established regularization strategies have been widely adopted, including Tikhonov regularization [43] and total variation regularization [44].

**Fig. 2:**
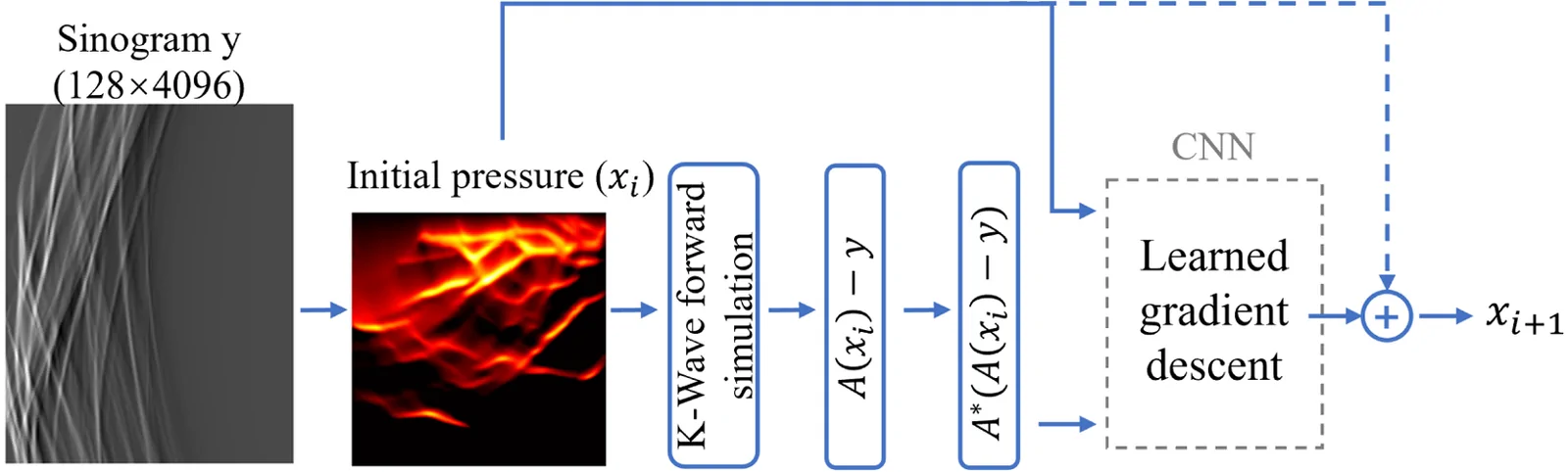
The framework of the model-based learning method.

### 3.2 Model-based learning

Model-based learning addresses the same optimization problem as the conventional MBI method. However, to avoid the need for sophisticated handcrafted parameter design, a learning-based module is introduced to replace the gradient descent process in the traditional MBI approach. This module is referred to as deep gradient descent or learned gradient descent [45], and it is commonly implemented using a convolutional neural network (CNN). The reconstruction pipeline of MBL is illustrated in Fig. 2, and can be mathematically formulated as:

$$x_{k+1}={\mathrm{CNN}}_{\theta_{k}}\left(x_{k},A^{*}\left(A\left(x_{k}\right)-y\right)\right),$$

wherek=0,1,…,K−1.

Here, K denotes the total number of iterations, and ${\mathrm{CNN}}_{\theta_{k}}$ represents the learned gradient descent component at the k-th iteration. A(⋅) is the forward operator, and $A^{*}\left(\cdot \right)$ is the adjoint operator. The variables $x_{k}$ and $x_{k+1}$ represent the reconstructed images at the k-th and (k+1)-th iterations, respectively. The initial image $x_{0}$ can be obtained either by applying the adjoint operator to the measured sinogram y (*i.e.*, $A^{*}y$) or by directly using a back-projection algorithm. At each iteration, the image generated from the previous step and the gradient information derived from the adjoint operator are jointly used as inputs to the learned gradient descent component at the k-th iteration. Given the ground truth (GT) image $x_{\mathrm{GT}}$, the objective function for ${\mathrm{CNN}}_{\theta_{k}}$ is defined as:

$$L_{\theta_{k}}={\left\|{\mathrm{CNN}}_{\theta_{k}}\left(x_{k},A^{*}\left(A\left(x_{k}\right)-y\right)\right)-x_{\mathrm{GT}}\right\|}_{2}^{2}.$$

**Fig. 3:**
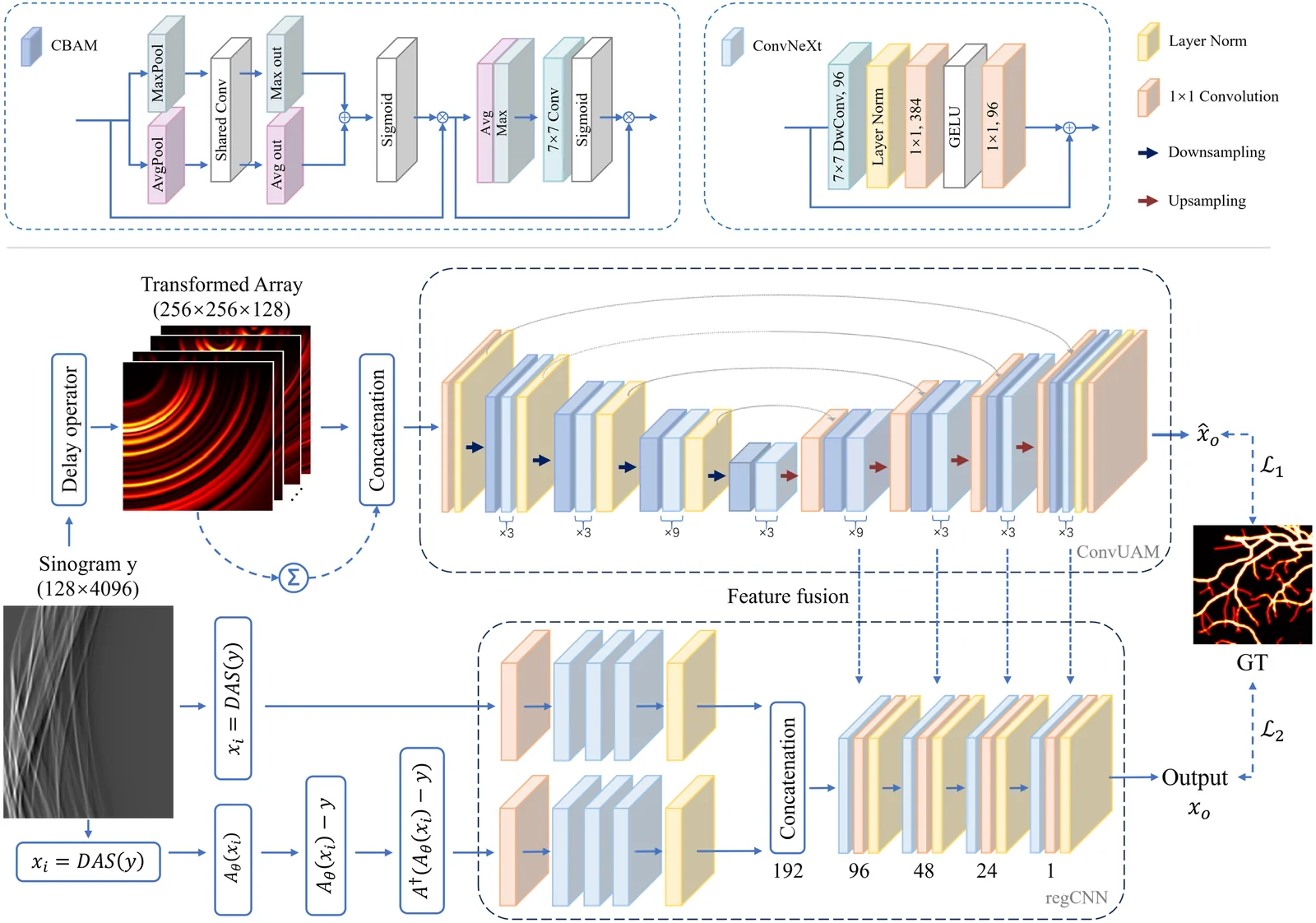
The framework of the proposed MB-JFF method. The detailed architecture of the joint feature fusion module is shown on the right side of the framework. The upper portion of this module delineates the principal components of our ConvUAM network, while the lower portion provides visualizations of the regCNN network.

## 4 Proposed method

### 4.1 Model-based joint feature fusion framework

Under the assumption of a homogeneous and lossless acoustic medium, we propose a novel model-based joint feature fusion framework, as illustrated in Fig. 3. The overall workflow of MB-JFF is composed of two primary stages: a feature extraction stage (the upper part of the framework) and a model-based learning stage (the lower part of the framework). These stages collectively transform the raw PA sinogram into its corresponding spatial representation, enabling robust PAT reconstruction. In contrast to the multiple iterative steps required by MBI and MBL methods, the proposed framework obtains high-quality reconstruction in just a single iteration.

In the first stage of the MB-JFF framework, the raw PA sinogram undergoes a delay operator to facilitate spatial–temporal mapping. Specifically, a Lookup Table (LUT) stores time-of-flight (ToF) values representing the acoustic propagation delays between each spatial pixel and each transducer element, determined by their Euclidean distances and the speed of sound [20], [22]. By indexing this LUT, raw time-domain sensor data are efficiently transformed into a 3D spatial representation, effectively mapping signals to their candidate spatial locations. The delay operator facilitates the transition between the time-series measurement domain and the spatial image domain, thereby enabling more effective feature learning in subsequent neural network processing. Following the delay operator, the resulting 3D array is input into a feature extraction neural network, which is termed ConvUAM. ConvUAM is designed to simultaneously extract low-level and high-level features from the transformed array, which are then passed to the second stage of the MB-JFF framework for further processing.

In the second stage, we employ delay-and-sum (DAS) beamforming to generate a low-quality initial image $x_{i}$ from the measured sinogram y. The DAS algorithm obtains the spatial distribution of initial pressure by applying time-delay corrections and direct summation to sensor signals. Here, $x_{i}$ is treated as a coarse image-domain estimate of the initial pressure distribution. The learned hybrid forward operator $A_{\theta }$ is then applied to this estimate to predict the sinogram that would be measured if $x_{i}$ were the underlying initial pressure distribution. The difference between the predicted sinogram and the measured sinogram, $A_{\theta }\left(x_{i}\right)-y$, forms a data-consistency residual. This residual measures the inconsistency between the predicted and actual measured data, thereby reflecting the accuracy of the image estimate $x_{i}$. Subsequently, a simplified adjoint operator $A^{†}$ (defined in Section 4.1.2) is applied to process the residual sinogram, and both $x_{i}$ and $A^{†}\left(A_{\theta }\left(x_{i}\right)-y\right)$ are fed into a neural network termed regCNN, which learns an update rule conditioned on the current image estimate and the residual. Notably, features extracted from the ConvUAM network are integrated into the intermediate layers of regCNN. The connection between ConvUAM and regCNN establishes a joint feature fusion module within our proposed framework, as illustrated in Fig. 3.

In the joint feature fusion module, ConvUAM produces an intermediate output ${\overset{ˆ}{x}}_{o}$ and simultaneously forwards its extracted features to regCNN, which processes the fused representations to produce the final output $x_{o}$. Both networks are jointly optimized using a weighted composite loss: $L=0.4\cdot L_{1}+0.6\cdot L_{2}$. $L_{1}$ and $L_{2}$ supervise the outputs of ConvUAM and regCNN, respectively, and they are both composed of a Mean Squared Error (MSE) loss ($L_{\mathrm{MSE}}$) and a Structural Similarity Index (SSIM) loss ($L_{\mathrm{SSIM}}$):

$$L_{1}=L_{\mathrm{MSE}\left({\overset{ˆ}{x}}_{o},\;x_{\mathrm{GT}}\right)}+0.5\cdot L_{\mathrm{SSIM}\left({\overset{ˆ}{x}}_{o},\;x_{\mathrm{GT}}\right)},$$

$$L_{2}=L_{\mathrm{MSE}\left(x_{o},\;x_{\mathrm{GT}}\right)}+0.5\cdot L_{\mathrm{SSIM}\left(x_{o},\;x_{\mathrm{GT}}\right)}.$$

The mathematical formulation for the $L_{\mathrm{SSIM}}$ component is as follows:

$$L_{\mathrm{SSIM}}=1-\mathrm{SSIM},$$

$$\mathrm{SSIM}\left(I_{p},I_{\mathrm{GT}}\right)=\frac{\left(2\mu_{I_{\mathrm{GT}}}\mu_{I_{p}}+\epsilon_{1}\right)\left(2\sigma_{I_{\mathrm{GT}}I_{p}}+\epsilon_{2}\right)}{\left(\mu_{I_{\mathrm{GT}}}^{2}+\mu_{I_{p}}^{2}+\epsilon_{1}\right)\left(\sigma_{I_{\mathrm{GT}}}^{2}+\sigma_{I_{p}}^{2}+\epsilon_{2}\right)}.$$

Here, μ is the local mean of image, σ is the local standard deviation, and $\sigma_{I_{\mathrm{GT}}I_{p}}$ is the local cross-correlation. Moreover, $\epsilon_{1}$ and $\epsilon_{2}$ are small constants to avoid division by zero. In the following part, we will detail the architecture of the two main networks in the joint feature fusion module, respectively.

#### 4.1.1 The ConvUAM network

For the feature extraction network, our research proposes ConvUAM, a U-Net-based [46] architecture that integrates ConvNeXt blocks [47] and Convolutional Block Attention Module (CBAM) [48] to process raw sensor data and extract multi-scale features. The overall architecture of ConvUAM follows a symmetric encoder–decoder structure, as illustrated in Fig. 3. The network accepts single-channel input and first applies a 1 × 1 convolutional projection layer to map the input into a 48-dimensional feature space. The encoder consists of four resolution-reduction stages. Each stage comprises a downsampling block followed by a stack of ConvNeXt blocks. The downsampling block performs spatial downsampling via a stride-2 2 × 2 convolution preceded by LayerNorm, and is immediately followed by a CBAM module to selectively recalibrate the downsampled feature maps. The four encoder stages progressively double the channel dimensions from 48 to 96, 192, 384, and 768, while halving the spatial resolution at each stage. The numbers of ConvNeXt blocks per stage follow the configuration {3,3,9,3}. Prior to each downsampling operation, the feature maps are retained as skip connections for the decoder. The decoder mirrors the encoder with four resolution-recovery stages. Each stage consists of an upsampling block followed by a stack of ConvNeXt blocks. The upsampling block first applies a 2 × 2 transposed convolution to double the spatial resolution and halve the channel dimension, followed by LayerNorm. The upsampled feature map is then concatenated with the corresponding skip-connected feature map from the encoder along the channel dimension, and a 1 × 1 convolution projects the fused representation to the target channel dimension. A CBAM module is subsequently applied to further refine the fused features. The channel dimensions in the decoder are progressively reduced from 768 back to 384, 192, 96, and 48. After the final decoder stage, a LayerNorm and a 1 × 1 output convolution produce the final feature map. This architecture leverages the multi-scale feature aggregation inherent in U-Net’s skip connections, while employing ConvNeXt blocks as the core backbone for hierarchical representation learning at each resolution stage. To further enhance feature discriminability, CBAM modules are strategically integrated within the encoder and decoder to act as adaptive recalibration mechanisms; these modules refine channel-wise and spatial responses, effectively prioritizing informative features following each resolution transition. Collectively, this synergistic integration facilitates effective multi-scale feature extraction from raw photoacoustic sensor data.

##### ConvNeXt block.

The remarkable performance of Vision Transformers across various visual tasks has prompted researchers to re-examine the innovative potential of pure convolutional neural networks. In this situation, ConvNeXt emerged as a modernized CNN architecture that systematically incorporates design principles from Transformer through four critical modifications. First, it employs depthwise convolutions that operate on each channel independently, mimicking the self-attention mechanism’s strategy of separating spatial and channel mixing. This allows the network to process spatial information within each feature channel before combining them. Second, it adopts an inverted bottleneck structure where the hidden dimension expands to four times the input dimension, enabling richer feature representations in the intermediate layers while maintaining parameter efficiency. Third, it utilizes large 7 × 7 convolutional kernels instead of the traditional 3 × 3 kernels, significantly expanding the receptive field to capture broader spatial context. Fourth, it replaces ReLU and BatchNorm with Gaussian Error Linear Unit (GELU) activation and LayerNorm, where GELU is a smooth nonlinear activation function that weights inputs according to their magnitude via a Gaussian cumulative distribution. These substitutions yield smoother gradient flow and more stable training dynamics. Together, all these targeted architectural innovations enable ConvNeXt to achieve competitive performance with Vision Transformers across diverse visual benchmarks, while retaining the computational efficiency and implementation simplicity inherent to standard convolutional neural networks.

##### CBAM block.

As shown in Fig. 3, the CBAM provides a lightweight mechanism for adaptive feature refinement through sequential channel and spatial attention modules. The channel attention module applies both average-pooling and max-pooling to capture complementary channel statistics, then processes them through shared convolutional layers to generate channel-wise attention weights that enable selective feature extraction. The spatial attention module pools along the channel axis and applies a 7 × 7 convolution to produce a spatial attention map highlighting critical regions. Through this sequential dual-attention design, CBAM enables sophisticated feature refinement without substantially increasing network complexity.

#### 4.1.2 The regCNN network

For the regCNN architecture in the second stage of MB-JFF, it also leverages ConvNeXt blocks to enhance its feature extraction and representation capabilities, enabling more effective capture of residual sinogram features. At the feature fusion stage, features from the ConvUAM network are first interpolated to match the size of those in regCNN, after which they are inserted into the intermediate layers of regCNN. Through the feature fusion process, ConvUAM and regCNN are combined to form a joint network architecture that effectively integrates their complementary strengths.

Compared to model-based learning, the learned gradient descent component in our proposed framework can be mathematically expressed as:

$$x_{o}=\mathrm{regCNN}\left(x_{i},A^{†}\left(A_{\theta }\left(x_{i}\right)-y\right),f\left(y\right)\right),$$

where $x_{i}$ and $x_{o}$ denote the initial image and the final reconstructed image, respectively, both with a size of 256 × 256 pixels. The term f(y) represents the features extracted by the ConvUAM network. $A_{\theta }$ refers to the learned hybrid forward operator introduced in Section 4.2. In the proposed MB-JFF framework, $A_{\theta }$ serves as a geometry-informed surrogate forward model in the model-driven residual branch. Specifically, $A_{\theta }$ maps the current image-domain estimate $x_{i}$ to a predicted sinogram, which is then compared with the measured sinogram y to form a data-consistency residual. $A^{†}$ denotes a simplified adjoint operator constructed by concatenating the outputs of delay operator and DAS beamforming. It is important to distinguish between the operator $A^{†}$ used in our MB-JFF framework and the operator $A^{*}$ employed in MBL methods. In MBL, $A^{*}$ represents the strict adjoint operator, which typically involves computationally expensive time-reversal simulation using the k-Wave toolbox [49]. While mathematically rigorous, this approach incurs substantial computational cost. In contrast, our $A^{†}$ adopts a simplified straight-line propagation model based on geometric propagation delay, thereby significantly reducing computational burden.

**Fig. 4:**
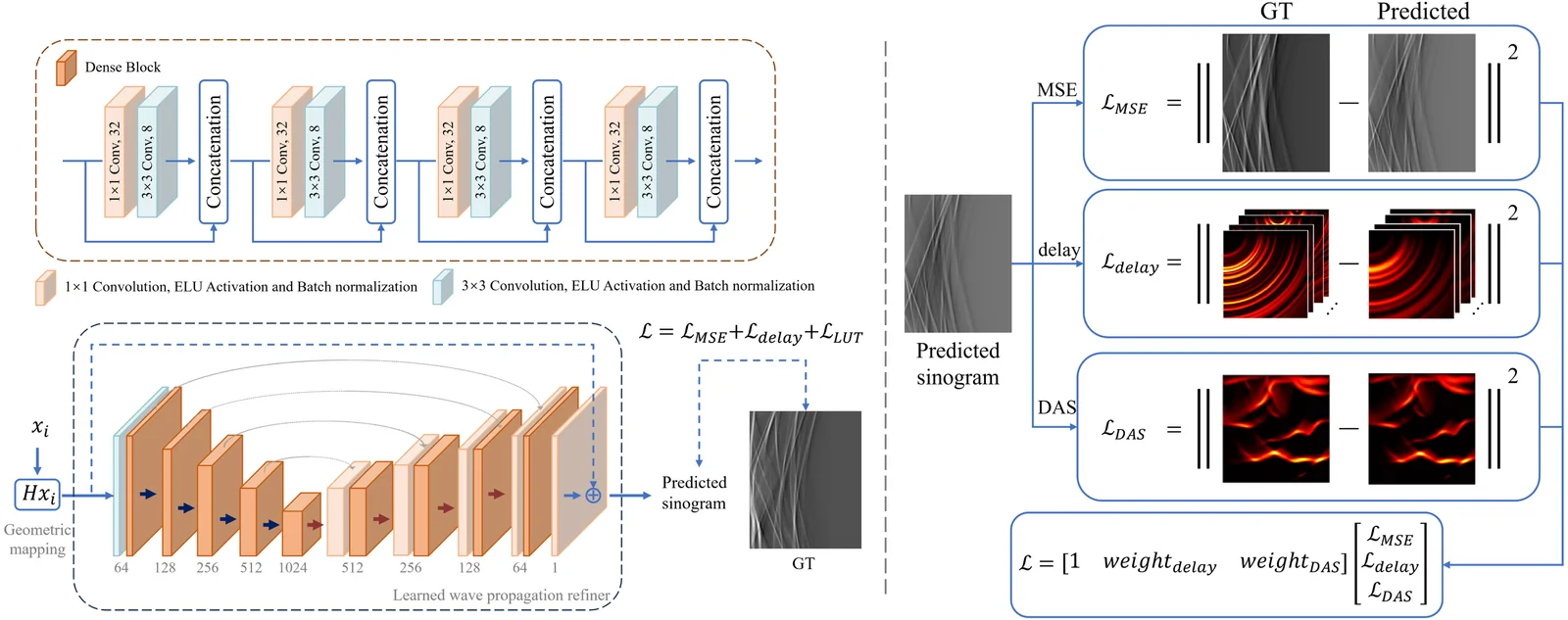
The schematic diagram of the proposed learned hybrid forward operator. The right side is the composite loss for the learned wave propagation refiner.

### 4.2 Learned hybrid forward operator

We propose a novel learned hybrid forward operator that balances computational efficiency and acoustic accuracy. Within the MB-JFF framework, this operator serves as a geometry-informed surrogate forward model that maps an image-domain initial pressure estimate to its corresponding PA sinogram. The predicted sinogram is then compared with the measured sinogram to obtain a model-driven residual sinogram for the joint feature fusion module. Unlike the FNO-based surrogate model [40], which employs a black-box architecture to directly predict the spatiotemporal evolution of the full-wavefield pressure, our strategy decouples the forward process into two distinct stages: global geometric mapping and local wave-propagation refinement. The overall strategy is illustrated in Fig. 4.

Specifically, for geometric mapping, we employ a simplified sparse physical matrix H, derived from a ray-based acoustic propagation model [14], to perform an efficient initial forward projection. This matrix serves as the backbone, ensuring that the initial sinogram strictly adheres to the fundamental geometric principles of PA wave propagation. The physical system matrix can be mathematically expressed as $H\in R^{\left(\mathrm{ML}\times N^{2}\right)}$, where M denotes the number of transducer elements, L denotes the total number of time steps, and $N^{2}$ denotes the size of the reconstructed image. In constructing the matrix H, a straightforward delay process is encoded: each pixel’s contribution is mapped to the corresponding time-delayed measurement at each transducer element. Specifically, for each detector-pixel pair (m,n), where m∈{1,…,M} and $n\in \left\{1,\ldots ,N^{2}\right\}$, the acoustic ToF is computed as: $\tau_{m,n}=d_{m,n}/c_{0}$. Here, $d_{m,n}={\left\|r_{s,n}-r_{d,m}\right\|}_{2}$ is the propagation distance, $r_{s,n}$ is the position of the nth source pixel, $r_{d,m}$ is the position of the mth detector element, and $c_{0}$ is the speed of sound. The matrix is then populated as: $H\left[\left(m-1\right)L+\ell_{m,n},\;n\right]=1/d_{m,n}$, $\ell_{m,n}=\mathrm{round}\left(\tau_{m,n}/\Delta t\right)$, where Δt is the temporal sampling interval, and all remaining entries are zero. Since H reduces the full wave propagation to a geometric ray approximation based on ToF, a dedicated learned refinement module is introduced to compensate for the resulting physical inaccuracies.

For the wave propagation refiner, a fully dense U-Net (FD U-Net) architecture is utilized, which was originally proposed by Guan et al. [50] and further utilized by DiSpirito et al. [51]. This architecture incorporates dense blocks in both the expanding and contracting paths of the U-Net structure. The detailed structure of the FD U-Net and the dense block is shown in the left part of Fig. 4. The dense block enables each layer to learn additional feature maps based on knowledge obtained from all preceding layers. Specifically, the input to each layer in the dense block consists of the concatenated outputs from all previous layers along with the original input to that layer. This design ensures that each layer only needs to learn refinements that either augment previous layers or diversify the collective feature set, thereby rendering the dense block of the FD U-Net applicable to the wave propagation refinement process. In this framework, the FD U-Net is no longer an image-to-image enhancement network; instead, it functions as a learned residual solver operating in the sinogram domain. The learned refinement module is trained to compensate for the wave propagation effects neglected by the ray-based approximation, transforming the geometrically projected initial sinograms into sinograms that more closely match those generated by full-wave numerical simulation.

The proposed hybrid architecture decouples the responsibilities of the forward model: the geometric prior encodes the spatial–temporal arrival structure of acoustic signals via ToF information, while the learned refiner captures the nuanced physical waveform characteristics, thereby bridging the gap between simplified geometric assumptions and complex wave propagation theory. Consequently, this new learned hybrid forward operator yields high-quality predicted sinograms that closely approximate the ground-truth outputs generated by highly accurate numerical simulations.

#### 4.2.1 Loss function

To train the proposed network, we design a composite loss function which is defined as follows:

$$L=L_{\mathrm{MSE}}+{\mathrm{weight}}_{\mathrm{delay}}\cdot L_{\mathrm{delay}}+{\mathrm{weight}}_{\mathrm{DAS}}\cdot L_{\mathrm{DAS}}.$$

Here, $L_{\mathrm{MSE}}$ denotes the MSE loss between the predicted sinogram and the GT, where the GT is generated through the forward simulation of the MATLAB K-Wave toolbox. This component is primarily employed to ensure the overall accuracy of the predicted sinogram. $L_{\mathrm{delay}}$ represents the MSE loss computed on the transformed arrays, which are obtained by applying the delay operator to both the predicted sinogram and the GT sinogram. This loss ensures that the result preserves consistency with GT in the transformed domain. Similarly, $L_{\mathrm{DAS}}$ is the MSE loss computed on the DAS beamforming results of the two sinograms. It helps evaluate the quality of the preliminary beamformed outputs and makes the predicted sinogram conform to the basic principles of photoacoustic wave propagation. The weights (${\mathrm{weight}}_{\mathrm{delay}}$ and ${\mathrm{weight}}_{\mathrm{DAS}}$) are hyperparameters that balance the contributions of these specific losses. By integrating these components into a unified composite loss, our proposed strategy yields high-quality outputs that closely approximate the GT, thereby enhancing both the fidelity of the results and the computational efficiency of the forward operator.

### 4.3 Network implementation

The proposed method was implemented through the PyTorch deep learning framework and conducted on a high-performance computing platform equipped with a NVIDIA GeForce RTX 4090 graphics processing unit. All the neural networks were optimized using the Adam optimizer, and the initial learning rate was set to $5\times 10^{-4}$. The training process was conducted for 300 epochs. Moreover, a cosine annealing learning rate scheduling strategy was employed to facilitate stable convergence, with the learning rate gradually decaying to approximately $1\times 10^{-7}$ at the final epoch. For the loss function configuration, a set of weights was employed to balance different loss components: ${\mathrm{weight}}_{\mathrm{delay}}$ and ${\mathrm{weight}}_{\mathrm{DAS}}$ were both set to 0.3.

The training procedure consists of two phases. First, the learned hybrid forward operator $A_{\theta }$ was trained to approximate the full-wave numerical forward solver. The training data for $A_{\theta }$ included two types of image-domain inputs: ground-truth initial pressure images and their corresponding limited-view DAS reconstructions. For each input image, the target output was the sinogram generated by applying k-Wave simulation to that respective image. Including DAS reconstructions in the training set allows $A_{\theta }$ to better handle the coarse image-domain estimates used in the model-driven residual branch of the MB-JFF framework. Second, the MB-JFF reconstruction network was trained to reconstruct the initial pressure image from the raw sinogram. In this stage, the ConvUAM and regCNN components were trained jointly, while the trained $A_{\theta }$ was fixed and used only to predict the sinogram from the image-domain estimate, from which the model-driven residual sinogram was computed by comparison with the measured sinogram.

## 5 Experiments

### 5.1 Data preparation

We utilized the k-Wave toolbox to generate the synthetic dataset for training. The transducer was configured as a 128-element linear transducer array with a uniform inter-element spacing of 0.3 mm. The frequency response of each transducer element was characterized by a center frequency of 2.5 MHz and a fractional bandwidth of 100%. The sampling frequency was set to 80 MHz, and the total number of time steps was 4096. The sound speed was assumed to be 1500 m/s, while the reconstructed region covered an area of 38.4 mm × 38.4 mm. For the k-Wave configuration, a computational grid of 256 × 256 points was employed, corresponding to a spatial grid spacing of 0.15 mm in both lateral and axial directions. This spatial discretization was selected to provide accurate full-wave simulations while maintaining computational efficiency for large-scale dataset generation. Publicly available fundus oculi vessel datasets, specifically DRIVE [52] and RAVIR [53], were employed as the initial pressure distributions. To increase the size and diversity of the training data, the vessel images were further augmented through segmentation and rotation operations. In total, 3256 samples were generated and randomly divided into training, validation, and test sets with a ratio of 8:1:1. The real phantom sinograms used for testing were acquired using a real photoacoustic imaging system with the same sensor configuration, as illustrated in Fig. 8(a). They were utilized to evaluate the performance of the proposed framework trained on simulated data.

For the in vivo experiments, we utilized the MSOT-Abdomen [21] dataset, which was acquired using the MSOT inVision 128 small-animal PAT system. The system was equipped with a nearly circular transducer array covering 270° and consisting of 128 detection elements, with a sampling frequency of 40 MHz. The radius of the transducer array was 40.6 mm. The detection elements had a center frequency of 5 MHz with an approximately 60% fractional bandwidth. For the k-Wave configuration, a computational grid of 816 × 816 points was employed with a spatial grid spacing of 0.1 mm. The reconstructed region of interest was set to 256 × 256. The data were obtained by scanning the abdomen of nude mice. Although the reconstructed images generated by the MBI algorithm using full-channel data did not provide complete 360° coverage, they still served as realistic reference images. To emulate a limited-view configuration of a curved transducer array, we extracted the sensor data from the middle 64 channels, corresponding to an angular coverage of 135°. A total of 575 samples were used for training, while 124 samples were reserved for testing.

**Table 1:** Experimental validation of the similarity between time-series sensor data generated by our proposed solver and the k-space PSTD solver.

Approaches	RMSE↓	PSNR${}_{\text{DAS}}↑$	SSIM${}_{\text{DAS}}↑$
Conventional Matrix	0.601367	5.4519	0.549016
Ours	0.01593	48.26476	0.99463

**Table 2:** Quantitative comparison of our proposed solver, the k-space PSTD solver, and a conventional matrix-based solver against the analytical solution for a simple Gaussian initial pressure distribution.

Approaches	RMSE↓	PSNR${}_{\text{DAS}}↑$	SSIM${}_{\text{DAS}}↑$
Conventional Matrix	0.11201	22.97531	0.56851
Ours	0.00436	56.73054	0.99834
k-space PSTD	0.00208	60.29969	0.99986

**Table 3:** Evaluation of the reconstruction performance of different methods under linear detection geometry.

Approaches	RMSE↓	PSNR↑	SSIM↑	LPIPS↓
BP	83.02489	9.84483	0.22418	0.44425
TR	81.42593	10.09046	0.47422	0.40267
MBI	69.49754	11.47112	0.51566	0.31093
U-Net [20]	34.36398	17.81467	0.86575	0.0885
AS-Net [29]	26.27617	19.31439	0.79958	0.05003
MSD-Net [26]	32.91396	18.22213	0.8689	0.0808
MBL [32]	23.51058	21.2634	0.91201	0.03976
Ours	**15.2661**	**25.26352**	**0.95169**	**0.01864**

**Fig. 5:**
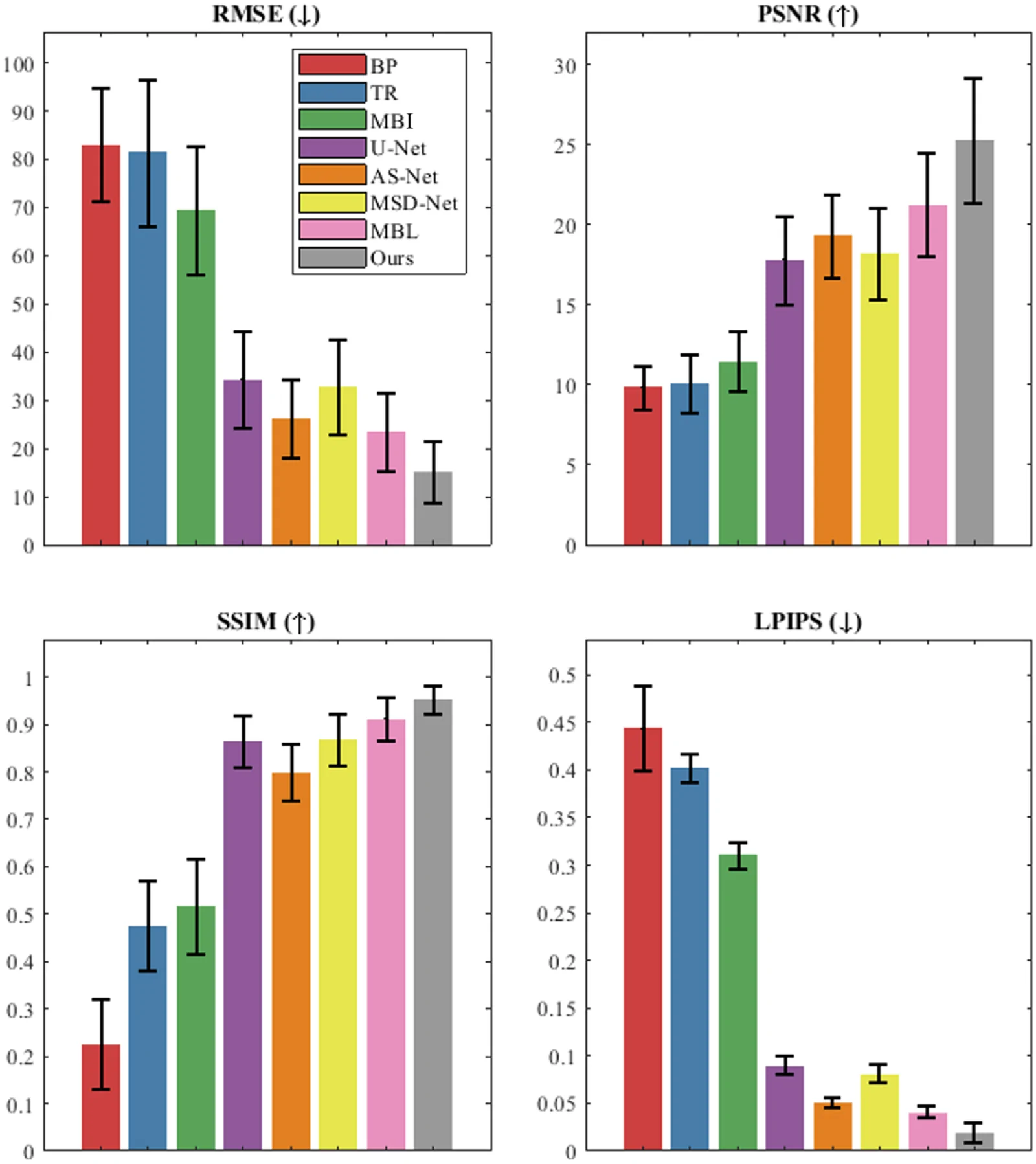
Comparison of the reconstruction performance of different methods under linear detection geometry.

### 5.2 Evaluation metrics

This study employs four metrics to systematically assess the fidelity and perceptual consistency of reconstructed images. Root Mean Square Error (RMSE↓) provides a foundational pixel-level precision quantification of reconstruction results. By measuring the absolute numerical divergence between generated and GT images, RMSE offers a rigorous assessment of image reconstruction accuracy. Peak Signal-to-Noise Ratio (PSNR↑) measures the ratio between the maximum possible pixel value of an image and the MSE between the reconstructed and reference images. PSNR is a widely used metric that quantifies the overall fidelity of a reconstructed image. Higher PSNR values indicate reduced noise and reconstructive artifacts, reflecting better image quality and lower distortion. While RMSE quantifies absolute numerical accuracy in the original pressure scale and thus reflects the quantitative accuracy of the recovered acoustic pressure magnitudes, PSNR evaluates reconstruction fidelity after independent normalization to a standardized dynamic range, thereby capturing structural quality and contrast recovery relative to background noise. The SSIM (↑) is a perceptual metric that evaluates image quality by comparing structural information between a reconstructed image and its reference. Unlike pixel-wise measures, SSIM assesses luminance, contrast, and structural similarity, providing a more accurate reflection of visual structural consistency. A higher SSIM value indicates greater structural fidelity and perceptual quality, reflecting exceptional morphological and textural similarity between the reconstructed and reference images. The Learned Perceptual Image Patch Similarity (LPIPS↓) [54] metric evaluates image similarity based on deep feature representations extracted from pretrained neural networks. Instead of comparing pixels directly, LPIPS measures perceptual differences in feature space, aligning more closely with human visual perception. Lower LPIPS values indicate higher perceptual similarity, meaning the reconstructed image appears more visually consistent with the reference.

### 5.3 Results and analysis

#### 5.3.1 Learned hybrid forward operator evaluation

First, we conducted experiments to validate that the proposed learned hybrid forward operator can generate sinograms that closely match those produced by the k-space PSTD solver. The results were summarized in Table 1, where the sinogram obtained from the k-space PSTD method was regarded as the GT. The experiments showed that the sinogram predicted by our solver closely matched the GT, with an RMSE as low as 0.01593. We further evaluated the DAS beamforming results obtained from both the predicted sinogram and the GT using PSNR and SSIM metrics. Both metrics exhibited extremely high values, indicating that the two DAS results were highly similar. This high similarity demonstrated that the predicted sinogram was not only consistent with the GT but also preserved the fundamental physical principles of photoacoustic wave propagation. In contrast, results obtained using the traditional matrix–vector multiplication method (as discussed in Section 4.2) performed significantly worse across all three metrics. To further validate the accuracy of the sinogram generated by the learned hybrid forward operator and assess the reliability of the k-space PSTD solver used as the reference, we additionally compared the conventional matrix-based solver, the proposed forward solver, and the k-space PSTD solver against an analytical solution in a homogeneous and lossless acoustic medium using a Gaussian initial pressure distribution. For the analytical reference, the initial pressure distribution was defined as a radially symmetric Gaussian source, $p_{0}\left(r\right)=\exp\left(-r^{2}/2\sigma^{2}\right)$, where r denotes the radial distance from the source center and σ controls the spatial extent of the Gaussian distribution (σ was set to 0.9mm). To ensure a fair comparison, a Gaussian bandpass filter corresponding to the transducer frequency response was applied to the analytical signal. This filtering step was introduced to match the frequency response settings used in the k-Wave simulations, thereby ensuring that the analytical solution incorporated the same bandwidth characteristics. As shown in Table 2, the sinogram generated by the learned hybrid forward operator closely matched the analytical solution, as evidenced by the consistently high quantitative metrics. Furthermore, excellent agreement was observed between the analytical solution and the k-space PSTD result, confirming that the k-space PSTD solver provided sufficient numerical accuracy under the spatial discretization adopted in this study.

Overall, these findings demonstrate that the sinogram generated by the matrix H serves as a coarse initial estimate obtained through a ToF-based mapping process. The learned refinement module can effectively compensate for physical inaccuracies neglected by the ray-based approximation. By combining these two stages, the proposed forward modeling strategy is able to generate final sinograms that closely match those produced by the conventional k-space PSTD approach. It should be noted that discrepancies between the experimentally measured data and those generated by the proposed learned hybrid forward operator may still arise from several factors that are not explicitly modeled in the current framework. These include acoustic attenuation in the propagation medium, the finite aperture and spatial directional response of practical transducer elements (in contrast to the idealized omnidirectional point receivers assumed in the k-Wave simulations), as well as electronic noise.

**Table 4:** Evaluation of the reconstruction performance of different methods under curved detection geometry on the unseen mouse abdomen dataset.

Approaches	RMSE↓	PSNR↑	SSIM↑	LPIPS↓
BP	151.06818	4.37898	0.27794	0.64744
TR	56.32984	13.17706	0.29728	0.31522
MBI	47.64920	14.65629	0.39075	0.30912
U-Net [20]	13.83959	26.68206	0.89038	0.06919
AS-Net [29]	23.50379	20.90482	0.83618	0.18132
MSD-Net [26]	15.18958	24.95558	0.85578	0.09615
MBL [32]	17.16799	23.48164	0.88361	0.08247
Ours	**8.48431**	**26.78494**	**0.93527**	**0.04453**

**Fig. 6:**
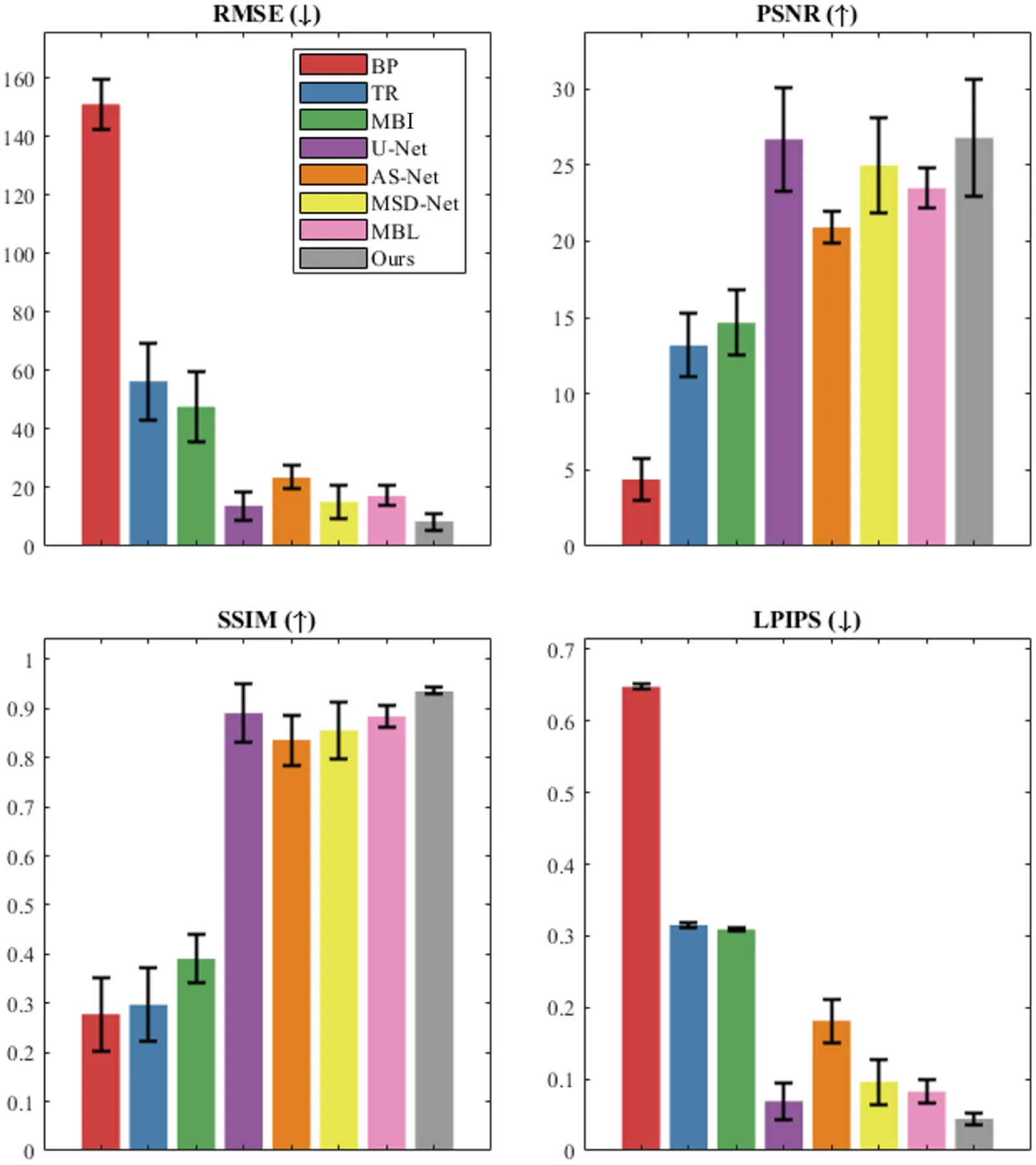
Comparison of the reconstruction performance of different methods under curved detection geometry.

**Fig. 7:**
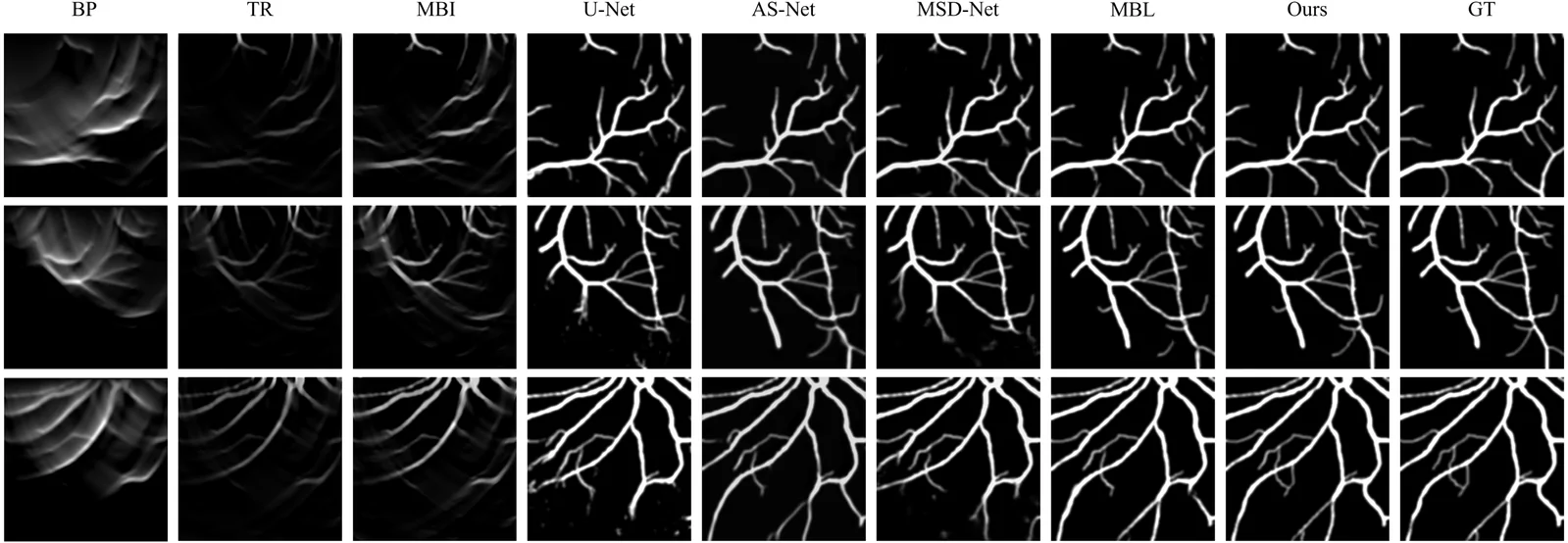
Visualization of photoacoustic image reconstruction using different methods under linear detection geometry scenario.

**Fig. 8:**
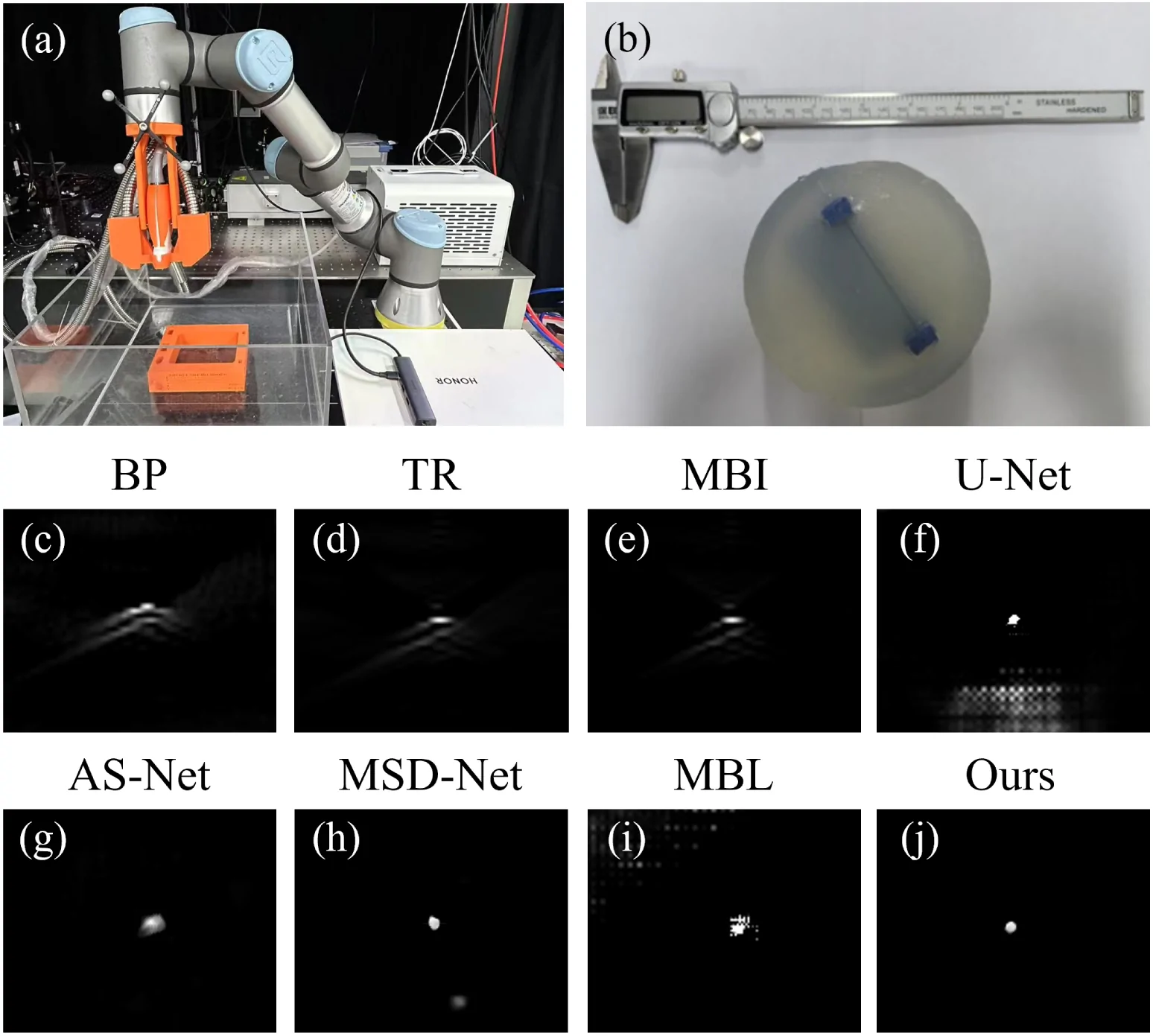
Visualization of photoacoustic image reconstruction using different methods under linear detection geometry. The time-series sensor data were acquired by scanning a pencil-lead cross-section with a real PAT system. (a) is the photograph of the real photoacoustic system, (b) is the photograph of the pencil lead phantom, and (c)–(j) are the reconstructed photoacoustic images obtained using different reconstruction methods.

**Fig. 9:**
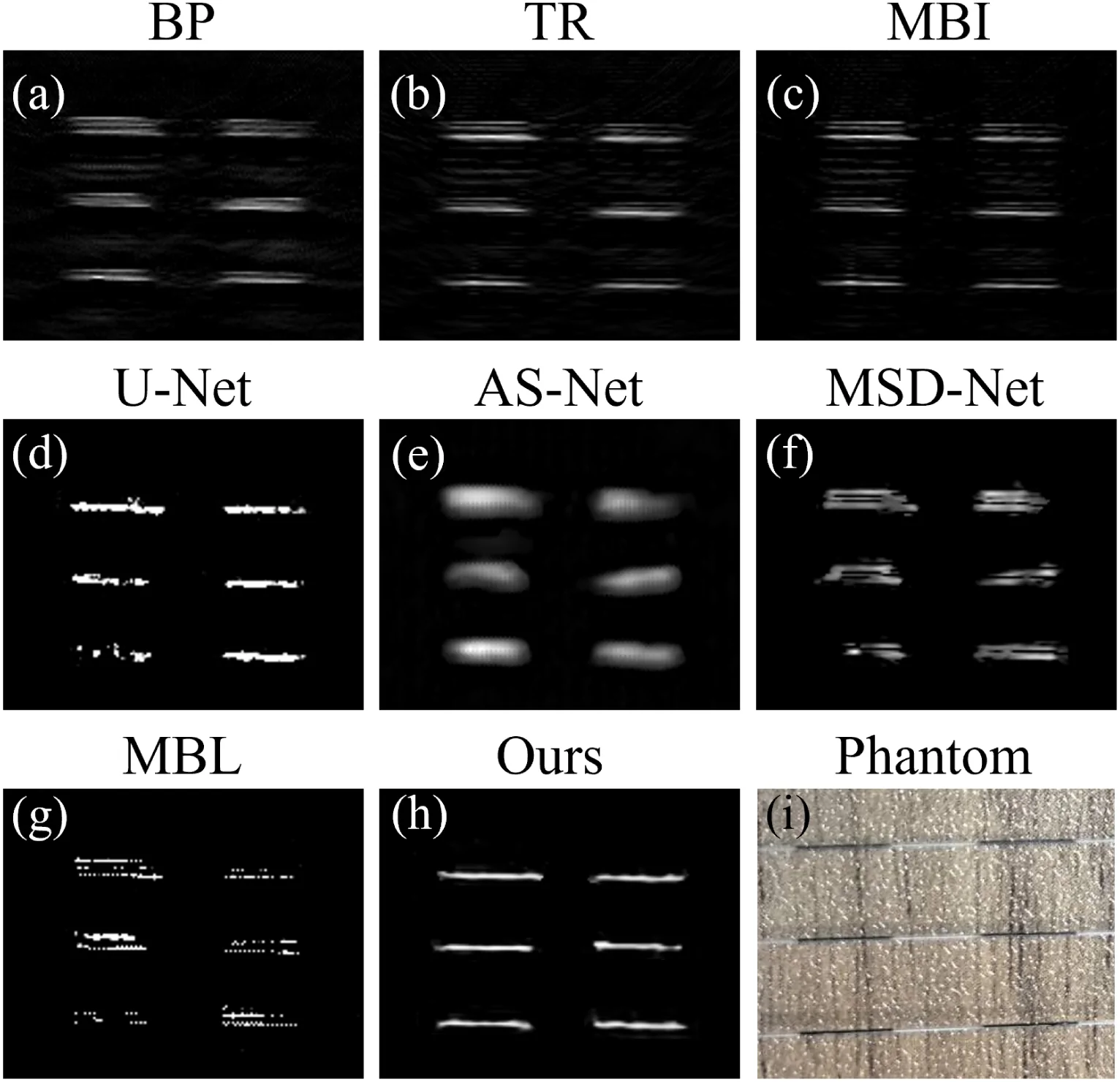
Visualization of photoacoustic image reconstruction using different methods under linear detection geometry. The time-series sensor data were acquired by scanning three partially darkened line segments with a physical photoacoustic imaging device. (a)–(h) are the reconstructed photoacoustic images obtained using different reconstruction methods, (i) is the photograph of the phantom.

#### 5.3.2 Reconstruction results

To further verify the effectiveness and advancement of the framework proposed in this study, we conducted a comprehensive comparison of our method against a broad range of existing approaches, including traditional, deep learning, and model-based learning methods. For the traditional category, all three conventional algorithms were evaluated (*i.e.*, back-projection, time reversal, and MBI algorithms). The MBI reconstruction estimated the initial pressure distribution by minimizing the discrepancy between the measured sinogram and forward model predictions via gradient-based optimization, where the forward and adjoint operators were implemented using the k-space PSTD solver and the time-reversal method, respectively, with TV regularization applied to suppress noise while preserving structural edges. For the deep learning category, we considered both well-established and recently proposed networks within the field as baselines, including the widely used network U-Net [20] and the specially designed AS-Net [29] and MSD-Net [26]. For the model-based learning category, we conducted experimental evaluations on the learned gradient descent neural network GE-CNN, which was proposed by Paul et al. [32]. Through this comprehensive comparative experimental design, we aimed to objectively verify the performance advantages of the proposed method across multiple dimensions, thereby laying a solid foundation for subsequent result analysis.

The experimental results obtained under different sensor configurations demonstrated that the MB-JFF method exhibited outstanding comprehensive performance in the PAT reconstruction task. For the experiments with a linear transducer array, the results were shown in Table 3 and Fig. 5, whereas for a curved transducer array, the results were presented in Table 4 and Fig. 6. Compared to all these methods, our proposed MB-JFF achieved superior performance across all four quantitative evaluation metrics (*i.e.*, RMSE, PSNR, SSIM, and LPIPS), indicating that the reconstructed images obtained through our proposed method exhibited superior fidelity. More specifically, the superior RMSE and PSNR values reflected high pixel-wise reconstruction accuracy and enhanced overall image quality, while the high SSIM score indicated that structural similarity was well preserved. Moreover, the superior LPIPS results demonstrated that our method produced images that exhibited high quality and fidelity from the perspective of human visual perception. The experimental results showed that MB-JFF effectively achieved both pixel-level accuracy and perceptual consistency in PAT reconstruction, outperforming other approaches across all metrics.

Fig. 7 presents the visual comparison of the reconstruction results for the vessel dataset under the linear transducer array. It is clear that the vascular network reconstructed by MB-JFF was clearer and more complete, with better continuity of the branching structure. Additionally, it maintained strong detail reconstruction capability in areas with thinner blood vessels. In contrast, the comparative methods exhibited varying degrees of distortion or structural loss in the vasculature. Fig. 8, Fig. 9 present the photoacoustic reconstruction results of two types of real phantoms: a pencil lead and three partially darkened line segments. For the pencil lead, which represents a typical point-like target, MB-JFF effectively suppressed artifacts and reconstructed a target that more accurately conformed to the point-like morphology of the pencil lead’s cross-section. The relatively weaker performance of the U-Net method in these real phantom experiments was likely attributable to the simulation-to-real domain shift. Since the network was trained entirely on simulated data, the presence of experimental noise in real phantom acquisitions may introduce discrepancies between the training and testing data distributions, thereby reducing its generalization capability. For the line segment phantom, our method demonstrated excellent performance in artifact suppression while maintaining straightness and preserving boundary sharpness. It accurately reconstructed the geometric features without the distortions or deformations observed in other methods. Fig. 10 presents a visual comparison of reconstruction results from mouse abdomen data acquired by a small-animal PAT system (a subset of channels was used for reconstruction to simulate a curved transducer array). In this experiment, the image reconstructed from full-sensor-channel data using the MBI method served as the reference. Under the limited detection angle of 135°, the reconstruction results exhibited severe quality degradation. Results from traditional algorithms exhibited noticeable artifacts and structural distortions. Learning-based approaches suppressed artifacts to some extent, but they still showed significant missing or distorted abdomen tissue structures. Model-based learning method yielded relatively better visual quality. However, it suffered from blurring and loss of fine details. In contrast, our proposed MB-JFF method demonstrated superior reconstruction performance under the same limited-view conditions. The reconstructed images not only showed a high degree of consistency with the full-channel reference in terms of overall visual effect, but also exhibited significant advantages in the following key aspects: major morphological structures were accurately preserved, while fine details were effectively recovered without the blurring or structural loss commonly seen in other methods. These results indicated that MB-JFF effectively compensated for the information loss caused by limited-view detection. Even in challenging in vivo imaging scenarios, it achieved high-quality reconstructions that most closely matched the full-channel reference.

**Fig. 10:**
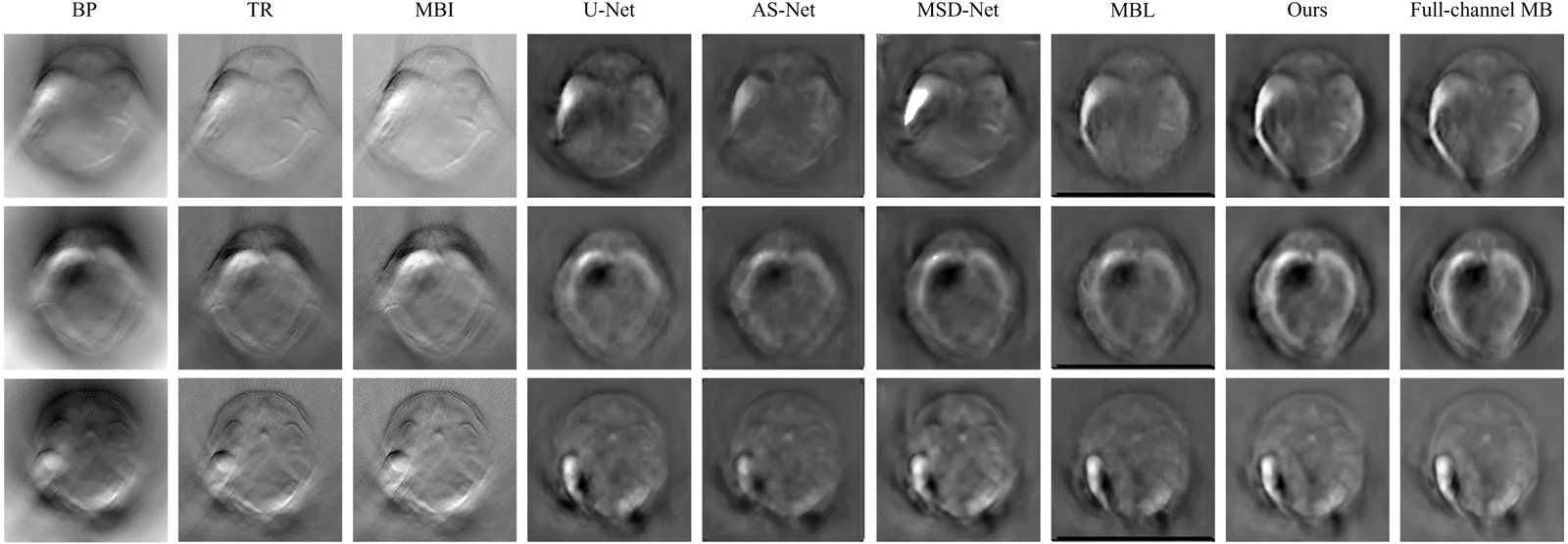
Photoacoustic image reconstruction of in vivo mouse abdomen using different methods under curved detection geometry.

**Table 5:** The experimental results of ablation study.

Approaches	RMSE↓	PSNR↑	SSIM↑	LPIPS↓
Ours-s1	21.79851	22.94267	0.92947	0.03051
Ours-s2	34.22286	17.90896	0.86665	0.09502
Ours	**15.2661**	**25.26352**	**0.95169**	**0.01864**

#### 5.3.3 Ablation study

To further verify the effectiveness of the proposed method, we conducted an ablation experiment on the vascular dataset. The specific designs were as follows: 1. “Ours-s1”: This configuration removed the second stage of MB-JFF. Under this setting, the reconstruction process retained only the first stage, which included the delay operator and the ConvUAM network used to extract features from the original PA signal. This experiment aimed to directly observe the impact on performance when the second stage is missing. 2. “Ours-s2”: This configuration removed the first stage and the feature fusion module of the framework, retaining only the complete process of the second stage. At this point, the system was regarded as an MBL framework, which leveraged the regCNN network as the learned gradient descent module. The forward operator was the novel solver that we proposed, and the simplified adjoint operator was the delay operator and the DAS beamforming, which was an approximate alternative to the strict adjoint operator. This experiment aimed to explore the performance when the feature fusion guidance from the original photoacoustic signal is missing. The experimental results presented in Table 5 demonstrated that the proposed overall MB-JFF framework achieved the best performance across all four key metrics. It verified the rationality of the overall design of MB-JFF and the complementarity between the two stages. The first stage of MB-JFF effectively extracted information from the original photoacoustic signal, while the second stage effectively utilized the information contained in the model-driven residual photoacoustic signal. The two stages worked together through feature fusion, which optimized the ability to restore details and suppress artifacts, thereby significantly improving the quality of photoacoustic reconstruction.

**Table 6:** Evaluation of the computational time of different methods.

Approaches	BP	TR	MBI	U-Net	AS-Net	MSD-Net	MBL	Ours
Time	3.237 ms	3.35s	97.46 s	8.237 ms	37.388 ms	15.545 ms	12.849 s	109.687 ms

#### 5.3.4 Computational efficiency evaluation

Additionally, to evaluate computational efficiency, we compared the runtime performance of various methods, as shown in the Table 6. The reported runtimes correspond to the mean reconstruction time per image, evaluated on the synthetic vessel test set, which consisted of 326 samples. For each method, the reconstruction time was measured individually for every test sample and then averaged across the entire test set. The comparative model-based methods, including MBI and MBL approaches, incurred substantial computational costs due to their reliance on complex forward simulations and iterative optimization. The MBL method required an average computational time of 12.849 s per image reconstruction, while the MBI method took 97.46 s. Even the TR method, which is non-iterative but involves wave-equation inversion, needed 3.35 s. This posed a significant challenge for applications requiring real-time or near-real-time processing. In contrast, our proposed MB-JFF method substantially reduced the computational burden, achieving an average runtime of 109.687 ms. This represented a remarkable reduction in processing time.

In summary, the experimental results demonstrated that MB-JFF achieved high-quality reconstruction performance while significantly reducing computational time. This improvement stemmed from the proposed framework, which enhanced reconstruction quality while reducing the number of required iterations, and from the newly designed learned hybrid forward operator strategy that minimized the physical simulation time. Moreover, by substantially accelerating model-based approaches, our method attained a well-balanced trade-off among reconstruction quality, generalization ability, and computational efficiency, offering a practical solution for real-time photoacoustic imaging applications.

## 6 Conclusion

In conclusion, this study proposes a novel model-based joint feature fusion framework and a new learned hybrid strategy for the forward operator. The MB-JFF approach utilizes a joint feature fusion module to improve feature extraction from both the raw sinogram and the model-driven residual sinogram, enabling a high-quality reconstructed image with only one iteration. By combining the advanced joint feature fusion module with forward model guidance, the framework effectively merges the strengths of data-driven and model-based methods. Furthermore, the novel learned hybrid forward operator leverages geometric mapping and a learned wave propagation refiner to efficiently generate realistic predicted sinograms while substantially reducing the computational cost relative to conventional numerical forward simulations. These innovations enable the MB-JFF framework to achieve high-quality image reconstruction with significantly reduced computational cost compared with other model-based methods, as confirmed by the comparative experimental results. The present validation was mainly conducted under the assumption of a homogeneous and lossless acoustic medium with a constant speed of sound. Therefore, the applicability of the proposed framework to heterogeneous media with large sound-speed variations remains to be investigated in future work. In addition, this study focuses on obtaining a single high-quality image estimate. Extending the proposed framework toward uncertainty quantification may further improve the interpretability and reliability of future real-time PAT reconstruction.

## CRediT authorship contribution statement

**Biru Zhang:** Writing – original draft, Validation, Software, Methodology, Investigation, Formal analysis, Data curation, Conceptualization. **Jiankun Wang:** Writing – review & editing, Resources, Project administration, Funding acquisition. **Max Q.-H. Meng:** Resources, Funding acquisition.

## Code availability

The implementation of the proposed MB-JFF framework is publicly available at: https://github.com/Crystal-Biru/MB-JFF-framework-for-PAT.

## Ethics declaration

This study was conducted in accordance with the following guidelines for animal welfare and/or reporting: Secondary analysis of a publicly available mouse abdominal imaging dataset; no animal experiments were performed by the authors. Ethics committee approval was not required. Ethics committee approval was not required because this study involved only secondary analysis of a publicly available animal imaging dataset, and no new animal experiments were conducted.

## Funding

This work was supported by 10.13039/501100001809National Natural Science Foundation of China under Grant 62561160114, Shenzhen Key Laboratory of Robotics Perception and Intelligence (ZDSYS20200810171800001), and the High level of special funds (G03034K003) from Southern University of Science and Technology, Shenzhen, China .

## Declaration of competing interest

The authors declare that they have no known competing financial interests or personal relationships that could have appeared to influence the work reported in this paper.

## Data Availability

Data will be made available on request.
